# Genome wide association in Spanish bread wheat landraces identifies six key genomic regions that constitute potential targets for improving grain yield related traits

**DOI:** 10.1007/s00122-023-04492-x

**Published:** 2023-11-13

**Authors:** Matilde López-Fernández, Julián García-Abadillo, Cristobal Uauy, Magdalena Ruiz, Patricia Giraldo, Laura Pascual

**Affiliations:** 1 Department of Biotechnology-Plant Biology, School of Agricultural, Food and Biosystems Engineering (ETSIAAB), Universidad Politécnica de Madrid (UPM), Madrid, Spain; 2 Department of Biotechnology and Plant Biology, Centre for Biotechnology and Plant Genomics (CBGP), Universidad Politécnica de Madrid (UPM), Madrid, Spain; 3 John Innes Centre, Norwich Research Park, Norwich, NR4 7UH UK; 4 Instituto Nacional de Investigacion y Tecnologia Agraria y Alimentaria (INIA), CSIC, Autovía A2, Km. 36.2. Finca La Canaleja, 28805 Alcalá de Henares, Madrid, Spain

## Abstract

**Key message:**

**Association mapping conducted in 189 Spanish bread wheat landraces revealed six key genomic regions that constitute stable QTLs for yield and include 15 candidate genes.**

**Abstract:**

Genetically diverse landraces provide an ideal population to conduct association analysis. In this study, association mapping was conducted in a collection of 189 Spanish bread wheat landraces whose genomic diversity had been previously assessed. These genomic data were combined with characterization for yield-related traits, including grain size and shape, and phenological traits screened across five seasons. The association analysis revealed a total of 881 significant marker trait associations, involving 434 markers across the genome, that could be grouped in 366 QTLs based on linkage disequilibrium. After accounting for days to heading, we defined 33 high density QTL genomic regions associated to at least four traits. Considering the importance of detecting stable QTLs, 6 regions associated to several grain traits and thousand kernel weight in at least three environments were selected as the most promising ones to harbour targets for breeding. To dissect the genetic cause of the observed associations, we studied the function and in silico expression of the 413 genes located inside these six regions. This identified 15 candidate genes that provide a starting point for future analysis aimed at the identification and validation of wheat yield related genes.

**Supplementary Information:**

The online version contains supplementary material available at 10.1007/s00122-023-04492-x.

## Introduction

Bread wheat (*Triticum aestivum* L.) is one of the major staple crops, providing about 20% of dietary calories and proteins (Shewry and Hey 2015). Thus, identifying new genes or favourable alleles controlling key breeding traits, like yield, is mandatory to develop high-yield varieties and ensure food security. Elucidating the genetic control of key breeding traits has been challenging, since they are mainly quantitative traits controlled by multiple quantitative trait loci (QTLs) and affected by environmental factors (Sehgal et al. 2017; Sukumaran et al. 2018). Advances in high-throughput sequencing technologies coupled with Genome Wide Association Studies (GWAS), based on linkage disequilibrium formed over generations, offer the possibility to map QTLs with high resolution (Zhu et al. 2008). These approaches have allowed the identification of multiple QTLs for agronomic and quality traits, as well as, for stresses responses in a wide range of crops, such as rice (Yano et al. 2016), barley (Alqudah et al. 2014), maize (Li et al. 2013) or soybean (Fang et al. 2017). In species with complex genomes, like wheat, association analysis has also been successful for dissecting the genetic architecture of key traits (see Saini et al. 2021).

In wheat, yield can be dissected into three principal components, including number of spikes per area, grain number per spike and weight grain (normally expressed as thousand kernel weight; TKW) (Liu et al. 2018). From them, TKW is the most stable and heritable parameter, and can be further divided into kernel size and shape traits (grain length, width, and area) (Gegas et al. 2010). In addition to TKW and grain traits, several other traits can affect yield, such as spikelets per spike, spike length or plant height (Wu et al. 2014). Reduced plant height, for example has proved to increase yield since the introduction of semi dwarf varieties in the Green Revolution. Additionally, phenological traits, such as days to heading and maturity, have proved their importance, since wheat must develop biomass and flower at optimal environmental conditions (Trethowan 2014). In the last decade, hundreds of QTLs for yield related traits have been reported in bread wheat. Some studies have even lead to the identification of candidate genes, like *TraesCS2D01G331100*, an orthologue of the rice *D11* gene contributing to grain length and width (Tekeu et al. 2021), or the cloning of genes controlling the studied trait, such as *TaGW8*, associated with kernel size and weight (Yan et al. 2019).

Lately, several authors have identified stable QTLs based on meta-analysis. Cao et al. (2020) defined 58 QTL-rich clusters related with TKW, kernel number per spike and spike number, located in all the wheat chromosomes except 3B. Liu et al. (2020) identified and validated 76 core Meta-QTL (MQTL) regions, in all wheat chromosomes, related with wheat yield and its component traits. Yang et al. (2021) summarized studies developed for yield related traits in irrigation and drought/heat-stressed environments, and identified 86 MQTL, some of them only in one of the environments. Finally, Ma et al. (2022) integrated their work with previous studies and identified 58 QTLs for kernel size related traits in 11 wheat chromosomes. Although, thousands of QTLs have been already identified, additional studies including non-previously screened variability, have the potential to identify new genes according to Malik et al. (2021).

One of the main requirements for GWAS has been the use of highly diverse populations, such as landraces, in order to capture the available genetic variability for the trait of interest (Kulwal and Singh 2021). Landraces have been adapted specifically to their region of origin through their evolution in local environments characterized by a wide range of biotic and abiotic conditions (Zeven 1998; Lopes et al. 2015). Thus, landraces represent an important source of genetic variability and have provided novel alleles for various agronomic, quality, biotic, and abiotic stress response traits (Azeez et al. 2018; Lopes et al. 2015). Moreover, landraces are traditionally grown with less inputs and have the potential to widen the gene pool of modern cultivars by adding underexploited variability in wheat breeding programmes (Nazco et al. 2012).

Spanish wheat landraces present high diversity due to the wide range of climatic conditions present in the Iberian Peninsula (Ruiz et al. 2018; Chacón et al. 2020). The Spanish National Plant Genetic Resources Centre (Centro de Recursos Fitogenéticos, CRF-INIA, CSIC, Madrid), maintains the national collection of Spanish bread wheat landraces composed of 522 accessions. This collection contains landraces from all Spanish regions where bread wheat was cultivated in the first half of the twentieth century. From this collection, a primary subset of 189 genotypes were selected based on collection site data (altitude, longitude, latitude) and morphological spike traits to represent the available diversity (Pascual et al. 2020a). Pascual et al. (2020b) genotyped this subset, and showed that landraces present higher genetic diversity than modern cultivars sown nowadays in Spain. Thus, these materials may include new variability non-previously screened, as showed in a previous GWAS study with Spanish durum wheat landraces where most of the marker-trait associations identified had not been previously described (Giraldo et al. 2016).

The aim of this study was to identify new genomic regions associated to yield-related traits, including also grain size and shape, and phenological traits in the 189 genotyped Spanish bread wheat landraces. For this purpose, a characterization of eleven yield-related traits in these landraces was performed along five seasons. The subsequent GWAS analysis identified genomic regions controlling these traits across environments. Moreover, we identified putative candidate genes inside associated genomic regions based on in silico expression analysis and functional annotation.

## Material and methods

### Plant material and phenotyping

In this study, a set of 189 bread wheat Spanish landraces (*Triticum aestivum* subsp. *vulgare* (Vill.)), already described in Pascual et al. (2020a, b) and López-Fernández et al. (2021) were analysed. The 189 genotypes were selected based on their collection site data (altitude, longitude, latitude) and morphological spike traits, to include all the agroclimatic (from cold sub-humid areas in the northern parts of Spain to warm semi-arid regimes in the southeast) and morphologic diversity found in a wider collection of 522 Spanish landraces of *Triticum aestivum subsp. vulgare* (Vill.) (Gadea 1954). This selection was the starting point for the construction of the Spanish bread wheat landraces core collection described in a previous study (Pascual et al. 2020a).

To obtain the phenotypic data, all landraces were sown during five consecutive seasons in an augmented design in plots of four rows per genotype (1 m long). In the 2016–2017 season, the accessions were sown in Alcalá de Henares (40°31′17, 8″ N, 3°17′33″ W, Madrid). In the following seasons (2017–2018, 2018–2019, 2019–2020, and 2020–2021), the accessions were sowed in the same conditions in the experimental fields of the ETSIAAB, Universidad Politécnica de Madrid (40º25’ N, 3º42’ W, Madrid). Daily meteorological data were recorded over the period of study (autumn 2016 to summer 2021) at nearby weather stations.

Phenotyping was conducted for a total of eleven traits, including: (i) grain traits: grain area (Ar), grain perimeter (Perim), grain major ellipse (Majell) and grain minor ellipse (Minell); (ii) yield-related traits: thousand kernel weight (TKW), grain number per spike (GrnSpk), number of spikelets per spike (SplN), spike length (SpkLng) and plant height (PH); and (iii) phenological traits: days to heading (DH) and days to maturity (DM). Some data were available from previous studies (Pascual et al. 2020a; López-Fernández et al. 2021) but phenotyping was completed in this work (see Table S1). DH, DM, PH, SpkLng and SplN were recorded in accordance with the International Board of Plant Genetic Resources (IBPGR 1985). Grain size and shape data (Ar, Perim, Majell, Minell) were obtained scanning at least 300 kernels using GrainScan software (Whan et al. 2014).

Statistical analysis was conducted using R v.4.0.3 (R Core Team 2022). Normality was tested by the Shapiro–Wilk test (*p*-value < 0.01), and significant traits were log transformed to achieve normality if possible (only GrnSpk was log transformed for the analysis). Mean, standard deviation, maximum and minimum values, and coefficient of variation were calculated for each trait by season. Correlations between years inside each trait and correlations among traits were calculated with Spearman coefficient (*p*-value < 0.05). Homocedasticity was checked using the Levene test. The effect of season, the genetic structure of the collection, and their interaction were evaluated with the Kruskal–Wallis (*p*-value < 0.05) and Wilcox tests (*p*-value < 0.05).

### Genetic analysis

High-throughput genotyping data for the set of 189 accessions were available from Pascual et al. (2020b). In this previous work, the accessions were genotyped by DArTseq GBS technology at SAGA (Genetic Analysis Service for Agriculture, Mexico City, Mexico). For this study, from the total 58,660 raw SNPs (Single Nucleotide Polymorphism) markers available, those with the same allelic profile, more than 10% of missing data, or MAF < 0.05 (Minimum Allele frequency) were filtered out. The remaining markers were subjected to BLAST search against the currently available *Triticum aestivum* genome REFseq v2.0 (Zhu et al. 2021); only markers located in the genome (BLAST E-value < 5e − 10 and sequence identity > 90%) were kept. The genetic structure of the 189 accessions was calculated in Pascual et al. (2020b) based on the DArT (presence/absence) markers. The set of 189 accessions was divided in four genetic subpopulations, from now on named pop1, pop2, pop3 and pop4.

Linkage disequilibrium (LD) among markers was calculated using TASSEL 5.0 (Bradbury et al. 2007). Pair-wise LD was measured using the squared allele frequency correlations *r*2 and the values were plotted by chromosome against the physical distance to determine how fast the LD decays. A LOESS curve was fitted to the plot. LD decay was estimated according to Remington et al. (2001).

### Genome-wide association study

Associations between phenotypic and genotypic data were detected using TASSEL 5.0 (Bradbury et al. 2007). A unique estimation of the phenotypic value was obtained by BLUES (best linear unbiased estimate) for the traits with a correlation between seasons higher than 0.5 in all the analysed seasons. For the remaining traits, associations were conducted independently per season. Associations were detected by a general linear model (GLM) including as a covariate the genetic structure (Q matrix). The obtained *p*-values for each MTA (Marker Trait Association) test were corrected by Bonferroni. For this purpose, the threshold was calculated dividing the standard *p*-value = 0.05 by the number of independent tests obtained with Tagger function of Haploview v4.2 software with *r*2 = 1 threshold (Barrett et al. 2005). LD blocks containing an association with the trait were defined as the chromosomic region containing all the markers in a LD > 0.3 (Alemu et al. 2021) with the associated marker. To do so the allele frequency correlations *r*2 between a significant marker and the markers located up and downstream were screened, when a marker presented *r*2 > 0.3 we moved to the next one, the marker that presented an *r*2 lower than 0.3 was considered as the end of the LD block. MTAs in the same LD block (or with overlapping end-star for their LD blocks) were considered to belong to the same QTL and grouped in Marker Trait Association Quantitative Trait Loci (MTA-QTLs). High-density MTA-QTLs regions were defined as the regions with single or overlapping MTA-QTLs, including more than 4 associated traits.

For high-density MTA-QTLs regions, the effect of days to heading was tested performing a statistic linear model using DH trait as a covariate:

$$y = x_{1} \omega + x_{2} M + \varepsilon ,$$

where $y$ was a vector with phenotypic values, $x_{1}$ was the vector with covariate values, $\omega$ was the estimate of covariate effect, $x_{2}$ was the vector with the genotypic values of the marker (0;1), $M$ was the estimate of the marker effect and $\varepsilon$ was the error.

### Identification of candidate genes

Gene annotation for the MTA-QTLs regions was obtained using the gene models for high-confidence genes reported for the wheat genome sequence *Triticum aestivum* genome REFseq v2.1 (Zhu et al. 2021) available at https://urgi.versailles.inra.fr/download/iwgsc/IWGSC_RefSeq_Annotations/v2.1/. The function of all the genes was obtained from *Triticum aestivum* genome REFseq v1.0 (IWGSC 2018) available at https://urgi.versailles.inra.fr/download/iwgsc/IWGSC_RefSeq_Annotations/v1.0/.

Expression of the genes coded inside the high-density MTA-QTLs regions was analysed in silico with the gene expression dataset of Azhurnaya spring wheat developmental time course experiment (Ramírez-Gonzalez et al. 2018; Borrill et al. 2016). Genes that did not reach an expression of 0.5 transcripts per million of sequences (TPM) in target stages and tissues (from tillering stage, “shoot apical meristem”; from full boot, “spike”; from spike, “spike 30%” and “spikelets 30%”; from anthesis, “anther” and “stigma ovary”; from milk grain stage, “glumes”, “lemma” and “grain”; from soft dough, hard dough and ripening, "grain"; and from dough, "endosperm") were filtered out.

To check the possible relationship between the traits and candidate genes, KnetMiner software (Hassani-Pak et al. 2021) was used, using as keywords “1000-grain weight" OR "Grain yield" OR "Grain size" OR "Grain width" OR "Grain number" OR "Grain weight" OR "Grain length", and as gene list the candidate gene names.

## Results

### Uncovering the phenotypic diversity in Spanish bread wheat landraces

To evaluate the phenotypic diversity in the set of 189 bread wheat landraces, this material was characterized for eleven traits (including grain traits, yield-related traits and phenological traits) during five seasons (Table 1). The highest variation, based on the coefficient of variation (CV) among accessions, was observed for SpkLng and TKW, and the smallest for phenological traits (DH and DM). Phenological traits showed a high diversity, with differences ranging up to 48 days in heading (DH) and up to 33 in days to maturity (DM). This diversity reflected the potential of the Spanish landraces for adapting to a high range of environments.

**Table 1:** Summary of the phenotypic data obtained

	Trait	Season	Mean ± SD	Min	Max	CV	*p-value*	
							Season	Pop
Grain Traits	Ar (mm)	2016–2017	14.61 ± 1.36	10.56	18.34	9.30	***	***
		2017–2018	17.04 ± 1.53	12.65	20.37	8.95		
		2018–2019	15.32 ± 1.36	12.00	18.58	8.87		
	Perim (mm)	2016–2017	20.19 ± 1.13	16.15	23.03	5.61	***	***
		2017–2018	20.87 ± 1.16	17.70	23.52	5.54		
		2018–2019	20.32 ± 1.10	17.04	22.72	5.41		
	Majell (mm)	2016–2017	6.74 ± 0.43	5.20	7.77	6.33	ns	***
		2017–2018	6.79 ± 0.43	5.46	7.87	6.36		
		2018–2019	6.82 ± 0.43	5.43	7.76	6.30		
	Minell (mm)	2016–2017	2.76 ± 0.16	2.31	3.17	5.71	***	ns
		2017–2018	3.20 ± 0.16	2.73	3.58	4.86		
		2018–2019	2.86 ± 0.13	2.52	3.14	4.69		
Yield-related Traits	TKW (g)	2016–2017	26.20 ± 3.99	14.98	39.14	15.22	***	*
		2017–2018	40.24 ± 5.01	27.03	52.03	12.46		
		2018–2019	27.19 ± 3.84	16.59	37.92	14.12		
		2019–2020	31.92 ± 4.02	20.00	40.36	12.58		
	GrnSpk	2017–2018	3.17 ± 0.25	2.33	3.80	7.74	***	***
		2018–2019	2.92 ± 0.39	1.70	3.89	13.23		
		2019–2020	3.43 ± 0.22	2.83	4.06	6.30		
	SplN	2016–2017	19.11 ± 2.04	14	24	10.68	***	***
		2020–2021	17.12 ± 2.75	10	24	16.04		
	SpkLng (mm)	2016–2017	117.03 ± 19.12	59	168	16.34	***	***
		2020–2021	99.94 ± 16.57	59	143	16.58		
	PH (cm)	2016–2017	88.27 ± 11.74	53	119	13.30	***	***
		2018–2019	102.84 ± 8.72	73	125	8.48		
		2019–2020	122.35 ± 11.27	85	148	9.21		
		2020–2021	96.95 ± 11.75	67	125	12.12		
Phenological Traits	DH	2016–2017	171.23 ± 7.01	150	188	4.09	***	***
		2017–2018	183.77 ± 5.77	163	197	3.14		
		2018–2019	146.47 ± 6.87	112	160	4.69		
		2020–2021	169.22 ± 8.71	142	187	5.15		
	DM	2016–2017	206.86 ± 3.37	197	216	1.63	***	**
		2018–2019	180.87 ± 7.63	164	197	4.22		
		2020–2021	199.55 ± 5.79	182	214	2.90		

Descriptive statistics, and effect of the season and genetic structure (subpopulations) of the set of landraces in each trait

*SD* standard deviation; *Min* minimum; *Max* maximum; *CV* coefficient of variation (%); *p-value < 0.05; ** *p*-value < 0.01; ***p-value < 0.001; *ns:* non-significant

*Ar* Area, *Perim* Perimeter; *Majell* major ellipse, *Minell* minor ellipse; *TKW* Thousand Kernel Weight, *GrnSpk* grain number per spike, *SplN* Spiklets per Spike, *SpkLng* spike length, *PH* plant height, *DH* days to heading, *DM* days to maturity

**Fig. 1:**
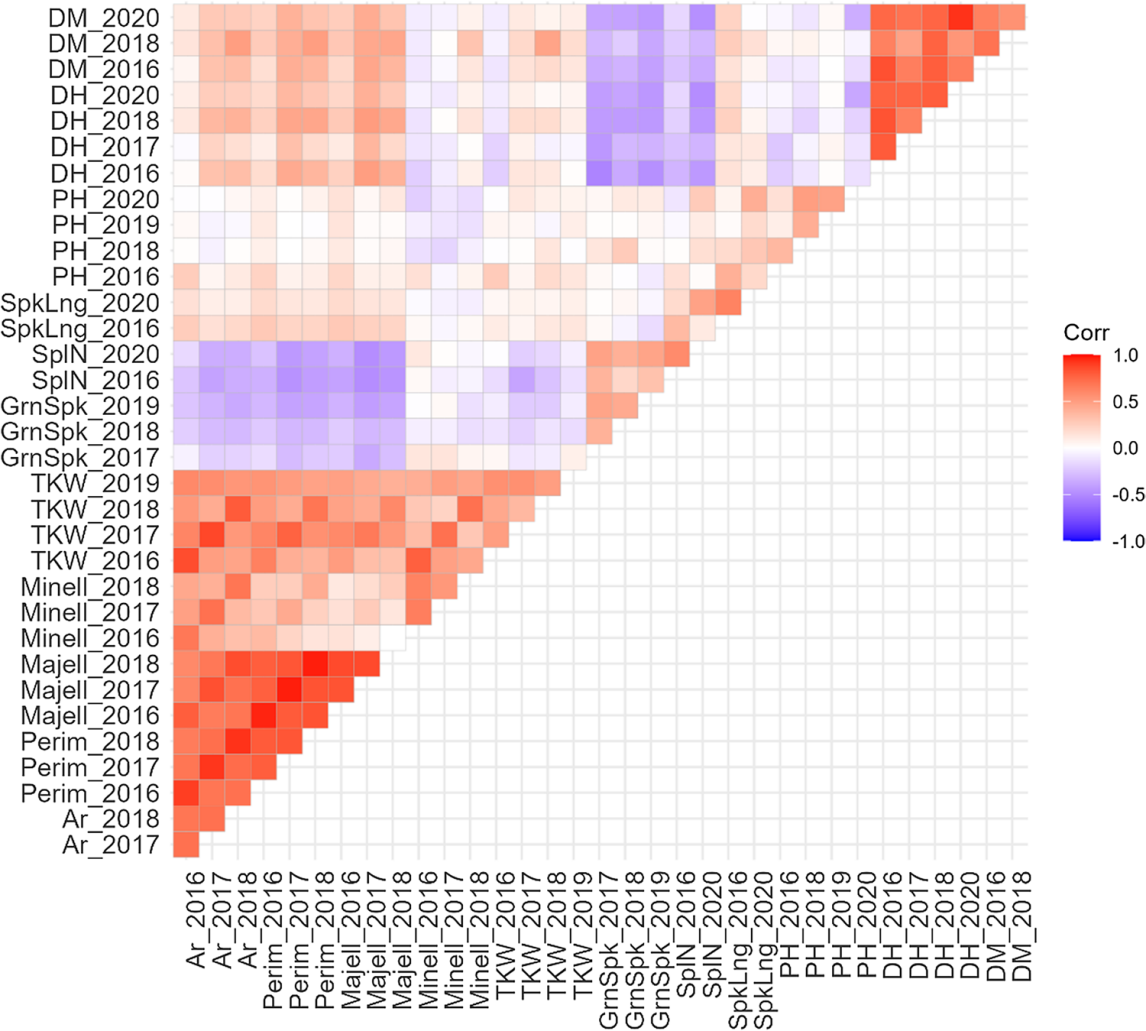
Correlations between traits and seasons. Positive correlation values in red gradient and negative values in blue gradient. Area: Ar; Perimeter: Perim; Major Ellipse: Majell, Minor ellipse: Minell; Thousand Kernel Weight: TKW; Grain number per spike: GrnSpk; Spiklets per Spike: SplN; Spike Length: SpkLng; Plant Height: PH; Days to Heading: DH; Days to Maturity: DM. The year indicates the sowing date for each season

As this set of landraces was clustered into four subpopulations (Pascual et al. 2020a), the effect of the genetic structure (pop) on the phenotype was evaluated. Significant differences were found for all the studied traits except Minell (Table 1). Besides, the environmental effect was also evaluated based on the different environments (seasons). A significant effect was found for all the studied traits, except Majell. Grain traits, TKW and DH values were higher on season 2017–2018, which was the wettest (Fig. S1). Although PH was not evaluated in that season, the highest PH values were found during the 2019–2020 season which was the second wettest. Moreover, DH, PH and TKW showed the lowest values on season 2016–2017, which registered the driest months during the grain filling period. To quantify this environmental effect, correlation analyses were carried out between seasons for each trait (Fig. 1). Positive to high positive correlations were observed for Ar, Perim, Majell, Minell, DH, DM, SpkLng and SplN. Thus, a unique phenotypic value across seasons was estimated for each of these traits through BLUES (Best Linear Unbiased Estimate). On the other hand, PH, GrnSpk and TKW showed low positive correlation values between seasons, due to the genotype x environment interaction, so each season phenotypes were kept separately for subsequent analysis.

Finally, correlations among traits were evaluated (Fig. 1). Grain traits (Ar, Perim, Majell and Minell) showed positive correlation values between them (except Majell with Minell), and with TKW, indicating the key role of the grain shape in grain weight. However, those traits were weakly and negative correlated with other yield-related traits (GrnSpk and SplN). DH and DM were positively correlated among them, as expected, but negatively correlated with GrnSpk and SplN (Fig. 1).

### Linkage disequilibrium along the chromosomes differed between homoeologous

High-throughput genotyping data for the set of 189 accessions had been previously reported at Pascual et al. (2020b). From the 58,660 raw SNP obtained on that study, a total of 4856 high-quality markers that could be located in Chinese Spring reference genome were selected for the analysis. Linkage disequilibrium (LD) among pairs of markers located in the same chromosome was calculated. The average square allele frequency correlation was *r*2 = 0.06 for the whole genome, ranging from 0.09 for chromosome 4B to 0.03 for chromosome 7D. The percentage of loci pairs showing a significant LD (*p* < 0.001) ranged from 28.56% for chromosome 1A to 9.41% for chromosome 4D. LD differed between homoeologous genomes with an average of 24.58% significant locus pairs (*r*2 mean = 0.07) corresponding to the B genome, 23.86% (*r*2 mean = 0.06) to the A genome and 11.82% (*r*2 mean = 0.04) to the D genome (Table S2). LD decay showed a similar trend for A and B genomes in all chromosomes, except for homoeologous group 4. For D genome chromosomes, LD decay was slower (Fig. S2A). The genome-wide half LD decay was 0.23 and the intersect of that value with the LD decay curve was at 1.3 Mb (Fig. S2B). Later, according to the HAPLOVIEW tagger function, it was determined that a total of 4476 independent test could be performed with the set of markers.

### Numerous marker trait associations were identified by GWAS

**Fig. 2:**
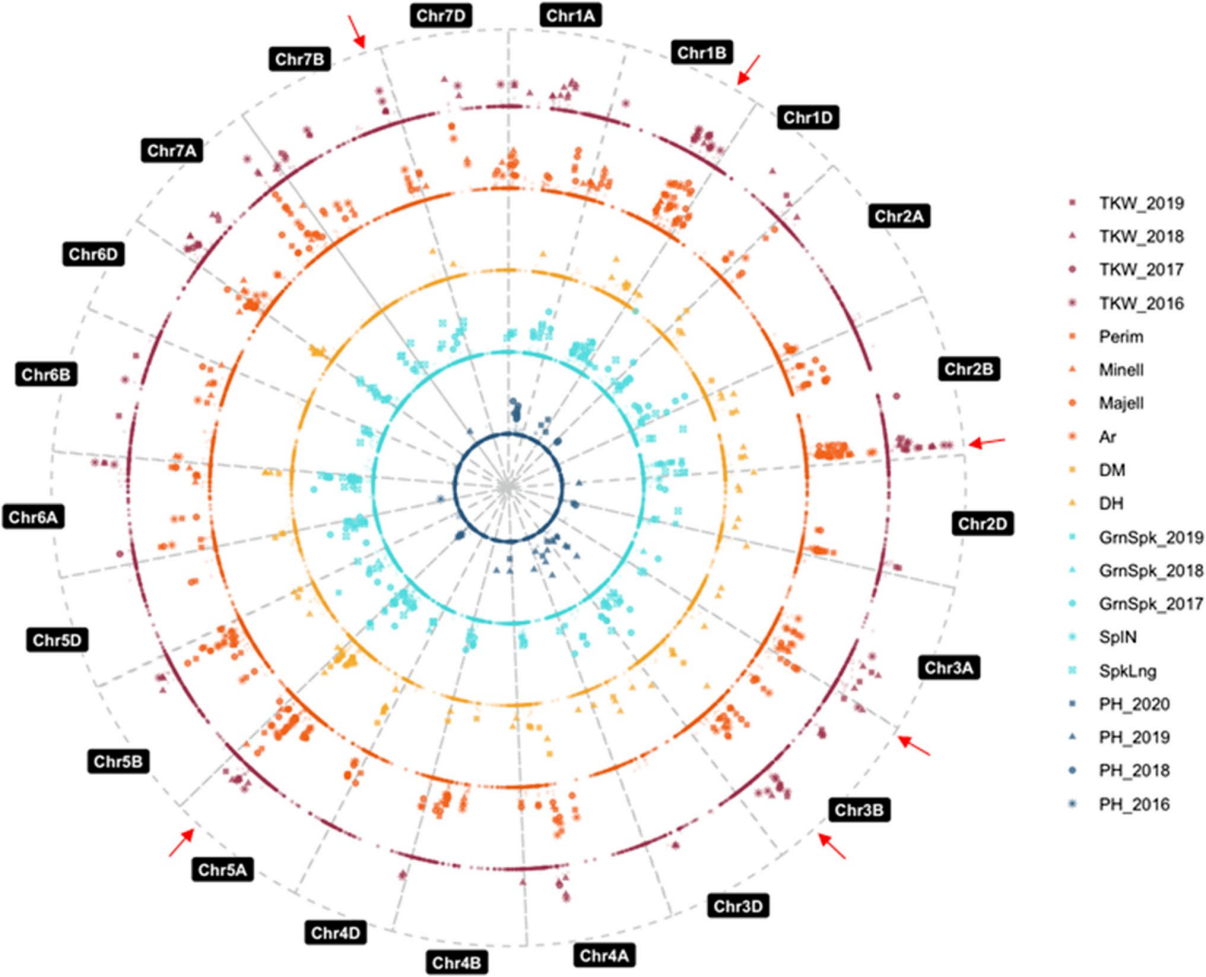
Manhattan plot including MTAs for Thousand Kernel Weight (TKW) in magenta; Area (Ar), Perimeter (Perim), Major Ellipse (Majell), Minor Ellipse (Minell) in orange; Days to Heading: DH; Days to Maturity: DM in yellow; Grain number per spike: GrnSpk; Spiklets per Spike: SplN; Spike Length: SpkLng in light blue; Plant Height: PH in dark blue.* P*-values in this figure where corrected by Bonferroni (that is, multiplied by the number of independent tests performed). Red arrows indicate the six genomic regions selected as the most promising in this study

**Fig. 3:**
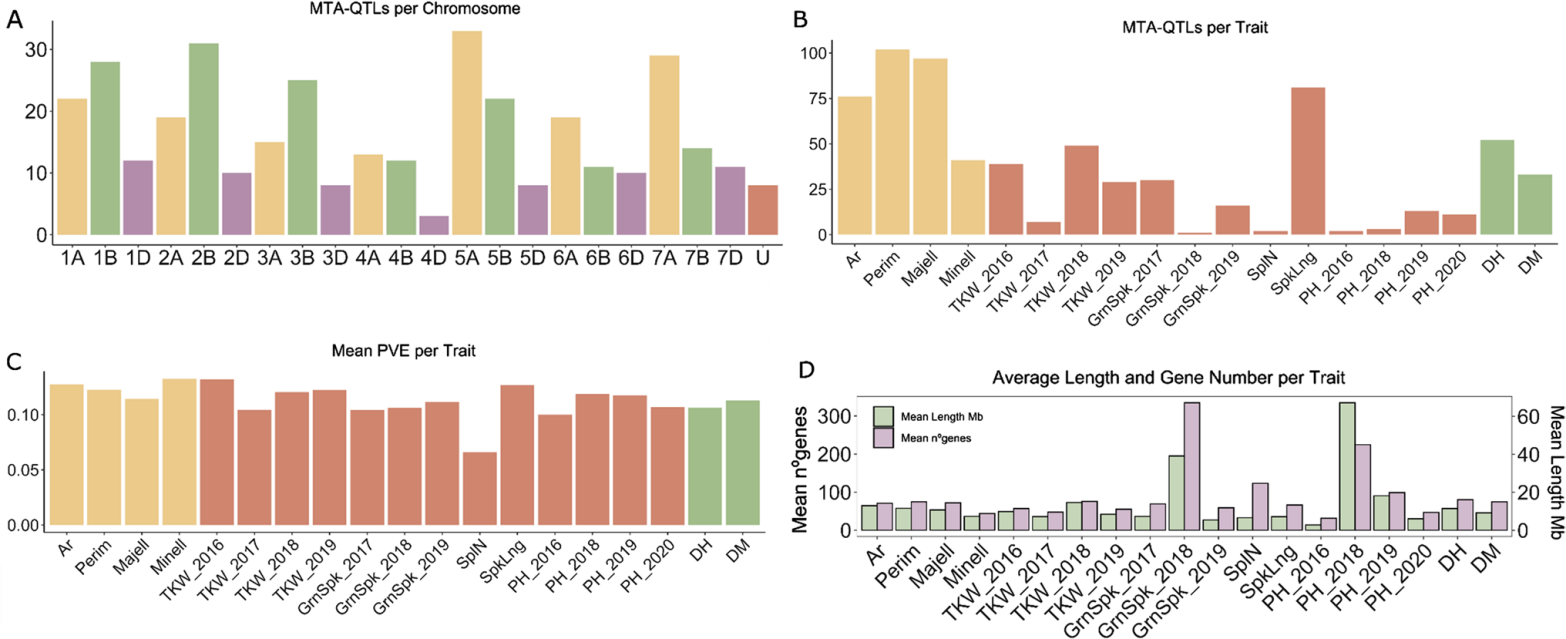
Summary of GWAS results. **A** Number of MTA-QTLs per chromosome.** B** Number of MTA-QTLs per trait. **C** Mean PVE per trait. **D** Average MTA-QTLs length and gene number per trait. Area: Ar; Perimeter: Perim; Major Ellipse: Majell, Minor ellipse: Minell; Thousand Kernel Weight: TKW; Grain number per spike: GrnSpk; Spiklets per Spike: SplN; Spike Length: SpkLng; Plant Height: PH; Days to Heading: DH; Days to Maturity: DM. The year indicates the sowing date for each season

With the aim of identifying the genomic regions associated with the evaluated traits, GWAS was performed. The analyses detected a total of 881 significant MTAs, involving 434 markers across the genome, as some markers were associated with more than one trait (Fig. 2, Table S3). The MTAs were distributed equally in the A and B genomes (~ 40%), and less in the D genome (~ 17%), consistent to the distribution of the whole set of SNP markers used for these analyses (Table 2). However, at the whole chromosome level, the distribution of MTAs was variable. Chromosome 5A showed the highest number of MTAs (112; 12.71%), despite not harbouring the highest number of SNP markers, whereas chromosome 4D showed the lowest (8; 0.91%), as expected since it is the smallest chromosome. Focussing on the traits, chromosome 4A, with only 3.52% of the total MTAs, harboured MTAs for the 11 traits studied. The number of MTAs associated with each trait ranged from 2 for SplN to 139 for Perim (Table S3). Finally, the mean percentage of phenotypic variance (PVE) explained per MTA was calculated, being its value similar for all traits, and ranging from 0.10 to 0.13, except for SplN (0.06) (Fig. 3c). Almost 70% of the MTAs showed a PVE lower than 0.12.

**Table 2:** Distribution of all associations identified along the wheat genome

Chr	Total markers	MTAs	MTA-QTLs	Associated traits
1A	291	51	22	Ar, Perim, Majell, Minell, TKW_2016, TKW_2018, GrnSpk_2017, GrnSpk_2019, SpkLng, PH_2018, DH
1B	277	81	29	Ar, Perim, Majell, Minell, TKW_2016, TKW_2017, TKW_2018, TKW_2019, GrnSpk_2017, GrnSpk_2019, SpkLng, PH_2020, DH, DM
1D	104	21	12	Ar, Perim, Majell, TKW_2018, TKW_2019, GrnSpk_2017, GrnSpk_2018, GrnSpk_2019, SpkLng, PH_2020, DH, DM
2A	303	32	19	Ar, Perim, Majell, TKW_2018, GrnSpk_2017, SpkLng, PH_2018, DH, DM
2B	346	88	31	Ar, Perim, Majell, Minell, TKW_2016, TKW_2017, TKW_2018, TKW_2019, GrnSpk_2017, GrnSpk_2019, SpkLng, DH, DM
2D	155	27	10	Ar, Perim, Majell, TKW_2019, GrnSpk_2017, GrnSpk_2019, SplN, SpkLng, PH_2019, DH
3A	315	27	15	Ar, Perim, Majell, Minell, TKW_2016, TKW_2018, TKW_2019, GrnSpk_2017, GrnSpk_2019, PH_2018, PH_2020, DH
3B	338	72	25	Ar, Perim, Majell, Minell, TKW_2016, TKW_2017, TKW_2018, TKW_2019, GrnSpk_2017, SpkLng, PH_2019, PH_2020, DH, DM
3D	106	21	8	TKW_2016, TKW_2018, GrnSpk_2017, SpkLng, PH_2019, PH_2020, DH, DM
4A	174	31	13	Ar, Perim, Majell, Minell, TKW_2016, TKW_2017, TKW_2018, GrnSpk_2019, SplN, SpkLng, PH_2019, DH, DM
4B	145	29	12	Ar, Perim, Majell, Minell, TKW_2018, SpkLng, PH_2019, PH_2020, DH, DM
4D	75	8	3	Ar, Perim, Majell, TKW_2016, TKW_2018, GrnSpk_2017
5A	328	112	33	Ar, Perim, Majell, Minell, TKW_2016, TKW_2018, TKW_2019, GrnSpk_2017, GrnSpk_2019, SpkLng, PH_2016, PH_2020, DH, DM
5B	295	43	22	Ar, Perim, Majell, Minell, TKW_2016, TKW_2018, GrnSpk_2017, GrnSpk_2019, SpkLng, DH, DM
5D	126	16	8	Majell, Perim, GrnSpk_2017, GrnSpk_2019, SpkLng, DH, DM
6A	207	45	21	Ar, Perim, Majell, Minell, TKW_2016, TKW_2017, TKW_2018, TKW_2019, GrnSpk_2017, SpkLng, PH_2016, DH, DM
6B	286	17	11	Ar, Perim, Majell, Minell, TKW_2016, TKW_2019, SpkLng
6D	121	30	10	Perim, Majell, Minell, SpkLng, DH, DM
7A	366	55	29	Ar, Perim, Majell, Minell, TKW_2016, TKW_2017, TKW_2018, TKW_2019, SpkLng, DH
7B	280	34	14	Ar, Perim, Majell, Minell, TKW_2016, TKW_2018, TKW_2019, PH_2019, SpkLng, DH
7D	144	21	11	Ar, Perim, Majell, Minell, TKW_2016, TKW_2018, SpkLng, DH
U	74	20	8	Ar, Perim, Majell, Minell, TKW_2016, TKW_2018, TKW_2019, SpkLng, PH_2019, DH, DM
Genome A	1984	353	152	
Genome B	1967	364	144	
Genome D	831	144	62	
Total	4856	881	366	

*Ar* Area, *Perim* Perimeter; *Majell* major ellipse, *Minell* minor ellipse; *TKW* Thousand Kernel Weight, *GrnSpk* grain number per spike, *SplN* Spiklets per Spike, *SpkLng* spike length, *PH* plant height, *DH* days to heading, *DM* days to maturity. The year indicates the sowing date for each season

To further determine the number of loci associated along the genome, the 881 MTAs were grouped into 366 Marker Trait Association Quantitative Trait Loci (MTA-QTL) based on the LD between flanking markers (Fig. 3, Table 2, Table S3). As for MTAs, chromosome 5A harboured the highest number (33), followed by chromosome 2B (31), while chromosome 4D harboured the lowest (3) (Fig. 3a and Table 2). Regarding the size of the MTA-QTLs, 165 (45%) included only one MTA, whereas the remaining 201 ranged from 2 (in 89 MTA-QTLs) to 19 MTAs. The average MTA-QTL physical length was 10.1 Mb (median 4.11 Mb), with 77.4% of them shorter than 10 Mb and 1.65% longer than 100 Mb. The smallest MTA-QTL, with only 20 kb, was located on chromosome 2B and the biggest one, with 214.22 Mb on chromosome 4A (Fig. 3d). The number of traits associated per MTA-QTL varied from 1 to 7. As expected, the traits with the highest number of MTAs (grain size traits (Ar, Perim and Majell) and SpkLng) were the ones with higher number of MTA-QTLs and the one with a lower number of MTA-QTLs (only 2) was SplN (Fig. 3b).

MTA-QTLs linked to the same trait when characterized in different environments are especially interesting and can be considered as stable QTLs. Stable QTLs could be target for the traits TKW, GrnSpk and PH analysed by season due to the lack of correlation between seasons. From the total of 89 MTA-QTLs identified for TKW (39 for season 2016–2017, 7 for season 2017–2018, 49 for season 2018–2019 and 29 for season 2019–2020) none of them was stable among all seasons. However, 10 were coincident in three seasons and 15 in two. For GrnSpk a total of 42 MTA-QTLs were identified (30 for season 2017–2018, 1 for season 2018–2019 and 15 for season 2019–2020) being only 4 stable on two seasons. Finally, for PH, 24 MTA-QTLs were detected (2 for season 2016–2017, 3 for season 2018–2019, 12 for season 2019–2020 and 11 for season 2020–2021), and also 4 were stable across 2 seasons. Besides stable QTLs, MTA-QTLs linked to several correlated traits also constitute a target that pinpoints genes with a possible pleiotropic effect. First, all co-localizing MTA-QTLs harbouring associations with grain size related traits were grouped. A total of 30 common MTA-QTLs for Ar, Perim and Majell, that could be considered key QTLs controlling grain size, were identified. For the two phenological traits DH and DM, 52 and 34 MTA-QTLs were detected, 17 common in both traits.

To identify candidate genes controlling the analysed traits, the genes inside the MTA-QTLs were analysed. The associations included a total of 25,373 genes according to IWGSC Wheat Refseq 2.1. The number of genes per MTA-QTL ranged from 0 to 656. The average number of genes per MTA-QTL was 71, with 9% of the MTA-QTLs contained less than 10 genes, and 7% more than 200 (Fig. 3d). The closest gene to the most significant marker for each trait within the MTA-QTLs and its predicted function was analysed (Table S3), and none of them matched known genes controlling the studied traits. However, several detected MTA-QTLs included or were close to key known genes. For example, MTA-QTL_4B.196 was located close to *VRN-B2*, MTA-QTL_5A.215 included *VRN-A1* and MTA-QTL_2D.115 was located close to *PPD-D1*, being all of them associated with DH and DM in cereal species (Fernández-Calleja et al. 2021; Chen et al. 2010; Yan et al. 2003; Welsh et al. 1973). Regarding to grain traits, MTA-QTL_6A.267 linked to TKW, co-localized with *TaGW2*. Also, as expected considering the Spanish landraces were collected before the Green Revolution, no MTA-QTLs for PH were located close to *RHT* genes on chromosomes 4B and 4D.

### Targeting high density MTA-QTL regions along the genome

Genomic regions associated to more than one trait could be interesting, specially to target genes that might help breeding for different traits. Thus, high-density MTA-QTL regions (from now on regions) were defined as a genomic interval including associations to four or more traits in only one MTA-QTL or in two or more overlapping MTA-QTLs. In total 46 regions were identified, most of them harbouring associations with grain traits and TKW (Table S4). Fourteen of those key regions were associated with DH. As it has been reported that DH might affect grain and yield related traits, DH effect on the associations identified in each region was tested. After this analysis, 33 regions remained associated to at least four traits (Table 3), including 6 regions where DH had been one of the associated traits, even though this association was no longer significant. In one of them, R5A.3, the size of the region was smaller.

**Table 3:** Description of the 33 selected genomic regions being associated with at least four traits

Region	MTA-QTLs	Chr	Region (Mb)	Associated traits	Genes	Closest gene	Annotation	Co-localize
R1A.1	1A.3, 1A.4	1A	34.02–42.08	Ar, Majell, Minell, TKW**	90	TraesCS1A03G0122300	RING/U-box superfamily protein	–
R1A.2	1A.16	1A	463.47–464.49	Perim, Majell, GrnSpk**, SpkLng	15	TraesCS1A03G0679900	Chaperone protein dnaJ	–
R1B.2	1B.32, 1B.33, 1B.34	1B	505.29–525.4	Ar, Perim, Majell, Minell, TKW***, SpkLng	110	TraesCS1B03G0804700	UPF0503 protein, chloroplastic	Ma et al. (2022)
R1B.4	1B.39	1B	573.83—578.27	Ar, Perim, Majell, TKW**	37	TraesCS1B03G0935200	Formin-like protein	Liu et al. (2020), Ma et al. (2022)
R1D.1	1D.60	1D	422.94–435.17	Ar, Perim, Majell, TKW*	145	TraesCS1D03G0780700	Formin-like protein	Ma et al. (2022)
R2B.1	2B.91	2B	230.57–235.85	Ar, Perim, Majell, SpkLng	29	TraesCS2B03G0551900	Protein phosphatase-2c, putative	Cao et al. (2020), Yang et al. (2021)
R2B.2	2B.92, 2B.93	2B	237.56–245.95	Perim, Majell, SpkLng, DM	60	TraesCS2B03G0568500	Mitochondrial pyruvate carrier	Cao et al. (2020
R2B.4	2B.100	2B	690.25–697.81	Ar, Perim, Majell, TKW**	64	TraesCS2B03G1224900	Multidrug resistance protein ABC transporter family protein	Liu et al. (2020)
R2B.6	2B.110, 2B.111	2B	752.7–757.03	Ar, Perim, Majell, Minell, TKW***	39	TraesCS2B03G1379000	Methylcytosine binding domain protein	Liu et al. (2020)
R2B.7	2B.112	2B	758.29–758.42	Ar, Perim, Majell, TKW**	4	TraesCS2B03G1392800	Anthocyanin 5-aromatic acyltransferase	Liu et al. (2020)
R3A.1	3A.131, 3A.132	3A	550.76–568.22	Ar, Perim, Minell, TKW**	132	TraesCS3A03G0766300	Purple acid phosphatase	–
R3B.1	3B.139, 3B.140, 3B.141	3B	12.61–16.82	Ar, Perim, Majell, TKW***, SpkLng	83	TraesCS3B03G0058000	Cytochrome P450, putative, expressed	Yang et al. (2021)
R3B.2	3B.145	3B	249.38–258.96	Ar, Perim, Majell, TKW***	62	TraesCS3B03G0503100	30S ribosomal protein S5	Liu et al. (2020)
R3B.3	3B.148, 3B.149	3B	517.99–535.10	Minell, GrnSpk, SpkLng, PH**	70	TraesCS3B03G0811300	GDSL esterase/lipase	Yang et al. (2021)
R3B.4	3B.155, 3B.156, 3B.157	3B	619.28–638.59	Ar, Perim, Minell, TKW****	112	TraesCS3B03G0984700	No note registered for this gen	–
R4A.2	4A.182	4A	612.61–614.01	Perim, Majell, SplN, DM	30	TraesCS4A03G0817700	DEAD-box ATP-dependent RNA helicase 52A	Cao et al. (2020), Ma et al. (2022), Yang et al. 2021)
R4D.1	4D.198	4D	12.32–25.31	Ar, Perim, Majell, TKW*	210	TraesCS4D03G0082900	Zinc-finger domain of monoamine-oxidase A repressor R1, putative	Cao et al. (2020), Liu et al. (2020), Yang et al. (2021)
R5A.1	5A.200	5A	49.59–136.02	Ar, Perim, Majell, DM	421	TraesCS5A03G0141900	Nitrate transporter 1.2	Cao et al. (2020)
R5A.2	5A.202, 5A.203, 5A.204, 5A.205	5A	459.1- 467.06	Ar, Perim, Majell, SpkLng, GrnSpk*	55	TraesCS5A03G0617500	Cyclin	Cao et al. (2020), Liu et al. (2020)
R5A.3	5A.212, 5A.213	5A	584.83–588.52	Ar, Perim, Majell, TKW*	61	TraesCS5A03G0931300	Beta-glucosidase	Cao et al. (2020), Yang et al. (2021)
R5A.4	5A.216, 5A.217, 5A.218, 5A.219, 5A.220	5A	590.25–596.91	Ar, Perim, Majell, Minell, TKW***	102	TraesCS5A03G0954900	Zinc-finger protein	Liu et al. (2020)
R5A.5	5A.221	5A	615.67–616.16	Ar, Perim, Majell, TKW*	9	TraesCS5A03G1017500	Blue copper protein	Liu et al. (2020)
R5A.6	5A.225	5A	673.15–675.35	Ar, Perim, Majell, TKW*, SpkLng	31	TraesCS5A03G1195900	Sugar transporter protein	Yang et al. (2021)
R5A.7	5A.229, 5A.230, 5A.231	5A	705.07–708.08	Minell, GrnSpk**, SpkLng, PH**	37	TraesCS5A03G1283300	Dirigent protein	Cao et al. (2020)
R5B.1	5B.246	5B	607.47–609.33	Ar, Perim, Majell, TKW*	28	TraesCS5B03G1055600	MD-2-related lipid recognition domain-containing protein/ML domain-containing protein	Ma et al. (2022)
R5B.3	5B.249, 5B.250, 5B.251, 5B.252	5B	645.07–662.73	Ar, Perim, Majell, DM	227	TraesCS5B03G1158800	Protein ELC	Ma et al. (2022)
R6A.1	6A.265, 6A.266	6A	66.41–84.24	Ar, Perim, Majell, TKW*, PH*	166	TraesCS6A03G0222800	Tryptophan synthase beta chain	Cao et al. (2020), Liu et al. (2020)
R6A.2	6A.271, 6A.272	6A	524.5–539.75	Ar, Perim, Majell, Minell	132	TraesCS6A03G0791700	TPR repeat-containing thioredoxin TTL4	Cao et al. (2020)
R6A.3	6A.278, 6A.279, 6A.280	6A	577.41–586.13	Majell, TKW*, GrnSpk*, SpkLng,	95	TraesCS6A03G0893300	Sugar transporter family protein, expressed	Cao et al. (2020), Yang et al. (2021)
R7A.1	7A.322, 7A.323, 7A.324	7A	617.38–621.40	Ar, Perim, Majell, Minell, TKW**	37	TraesCS7A03G1031200	Serine/threonine-protein kinase	Liu et al. (2020), Ma et al. (2022)
R7B.1	7B.340	7B	234.61–248.39	Ar, Perim, Minell, TKW***	61	TraesCS7B03G0447700	Glutamate dehydrogenase	Ma et al. (2022)
R7B.2	7B.343	7B	688.21–690.48	Ar, Perim, Majell, TKW***	17	TraesCS7B03G1115100	tumour necrosis factor receptor family protein	Liu et al. (2020
R7D.1	7D.352, 7D.353, 7D.354	7D	221.8–372.34	Ar, Perim, Majell, Minell, TKW**	461	TraesCS7D03G0643100	Defective in cullin neddylation protein	Cao et al. (2020), Liu et al. (2020), Ma et al. (2022)

*Ar* Area; *Perim* Perimeter; *Majell* Major Ellipse; *Minell* Minor ellipse; *TKW* Thousand Kernel Weight; *SpkLng* Spike Length. For TKW, number of * indicate the number of years for which associations were found

**Fig. 4:**
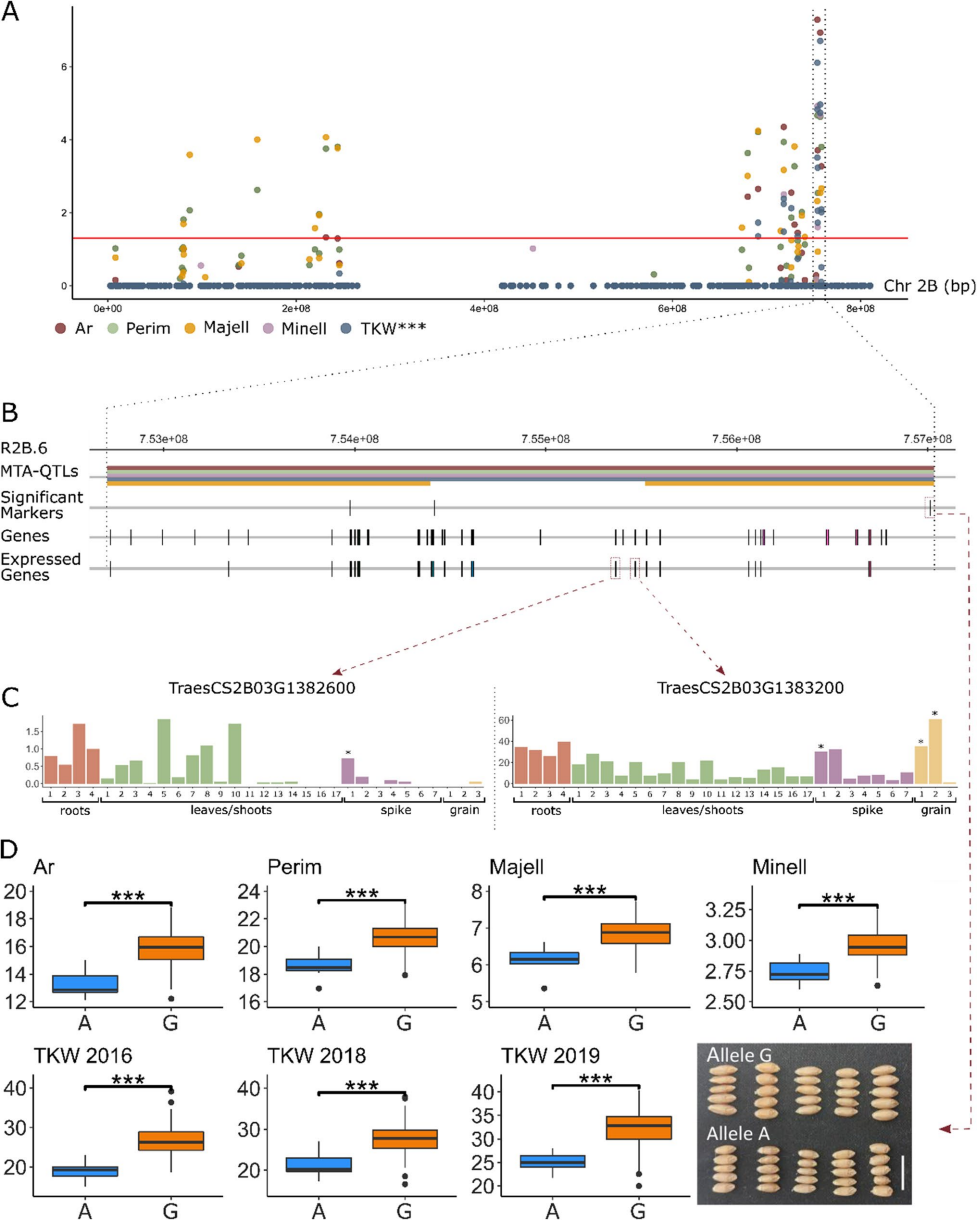
Details of the genomic region including the most significant MTA with TKW (R2B.6). **A** Manhattan plot for chromosome 2B, including MTAs for Area (Ar), Perimeter (Perim), Major Ellipse (Majell), Minor Ellipse (Minell) and Thousand Kernel Weight (TKW; number of * indicate the number of years for which associations were found). *P*-values in this figure where corrected by Bonferroni (that is, multiplied by the number of independent tests performed). **B** Zoom of R2B.6. **C** Putative candidates and their expression values. * indicates expression > 0.5 TPM (For roots 1:radicle, 2: roots, 3: root apical meristem, 4: axillary roots; for leaves/shoots 1:coleoptile, 2: stem axis, 3: first leaf sheath, 4: first leaf blade, 5: shoot apical meristem, 6: third leaf blade, 7: third leaf sheath, 8: fifth leaf sheath, 9: fifth leaf blade, 10: shoot axis, 11: flag leaf blade, 12: leaf ligule, 13: flag leaf sheath, 14: Internode #2, 15: peduncle, 16: fifth leaf blade senescence, 17: flag leaf blade senescence; for spike 1: spike, 2: spikelets, 3: awns, 4: glumes, 5: lemma, 6: anther, 7: stigma & ovary; for grain 1: grain, 2: endosperm, 3: embryo proper, according to www.wheat-expression.com; Ramírez-González et al. 2018; Borrill et al. 2016)). **D** Average trait value according to the allele carried by the accessions in the most significant MTA. The picture illustrates the differences on grain size between ten landraces, five carrying allele A (BGE001942, BGE018217, BGE023723, BGE001983, BGE002012) and five allele G (BGE001945, BGE015402, BGE023725, BGE003236, BGE003156)

As TKW and grain traits (Ar, Perim and Majell) represent a cornerstone for breeding, out of the 33 regions described previously, the six that were associate with these traits (TKW in three seasons) were selected as the most promising ones (Fig. 2, Table 4). For them, the effect of the allele carried by each accession, at the most significant MTA according to GWAS, in the average values of the associated traits was explored (Fig. S3). Region R2B.6 included the most significant MTA for TKW (Table S3, Fig. 4a and b), for this marker, the accessions carrying allele G presented an increase of 19.40% for Ar, 10.46% for Perim, 10.88% for Majell, and for TKW an increase up to 41.32% on season 2016–2017, 30.79% on season 2018–2019 and 29.30% on season 2019–2020 (Fig. 4d, Fig. S3).

**Table 4:** Description of the six selected genomic regions

Region	MTA-QTLs	Chr	Region (Mb)	Associated traits	Total Genes	Expressed genes	Putative candidates	Annotation
R1B.2	1B.32, 1B.33, 1B.34	1B	505.29–525.4	Ar, Perim, Majell, Minell, TKW***, SpkLng	110	91	*TraesCS1B03G0827400* *TraesCS1B03G0803000* *TraesCS1B03G0817400*	*E3 ubiquitin-protein ligase* *MYB transcription factor* *Myb-like protein*
R2B.6	2B.110, 2B.111	2B	752.7–757.03	Ar, Perim, Majell, Minell, TKW***	39	23	*TraesCS2B03G1383200* *TraesCS2B03G1382600*	*RNA binding protein* *Transcription factor protein*
R3B.1	3B.139, 3B.140, 3B.141	3B	12.61–16.82	Ar, Perim, Majell, TKW***, SpkLng	83	55	*TraesCS3B03G0058000* *TraesCS3B03G0054900* *TraesCS3B03G0055300* *TraesCS3B03G0055900*	*Cytochrome P450* *Receptor-like protein kinase* *Receptor-like protein kinase* *Receptor-like protein kinase*
R3B.2	3B.145	3B	249.38–258.96	Ar, Perim, Majell, TKW***	62	51	*TraesCS3B03G0504300* *TraesCS3B03G0496600*	*NAC domain protein* *WD-repeat protein*
R5A.4	5A.216, 5A.217, 5A.218, 5A.219, 5A.220	5A	590.25–596.91	Ar, Perim, Majell, Minell, TKW***	102	77	*TraesCS5A03G0945200* *TraesCS5A03G0956000*	*Protein kinase family protein* *Kinase family protein*
R7B.2	7B.343	7B	688.21–690.48	Ar, Perim, Majell, TKW***	17	11	*TraesCS7B03G1114600* *TraesCS7B03G1112900*	*Ubiquitin* *F-box family protein*

*Ar* Area; *Perim* Perimeter; *Majell* Major Ellipse; *Minell* Minor ellipse; *TKW* Thousand Kernel Weight; *SpkLng* Spike Length. For TKW, number of * indicate the number of years for which associations were found

To dissect the genetic cause of the observed associations, the function fo the 413 genes located inside these 6 regions was studied. First, these genes were classified based on their GO terms (Fig. S4). According to Biological Process, 162 and 143 genes were included in “cellular process” and “metabolic process”, followed by biological regulation (52 genes). Regarding Molecular Function, the main categories with 167 and 146 genes were “catalytic activity” and “binding”, followed by “transferase activity” (80 genes). Second, to select putatives candidate genes, the genes were filtered by relevant tissue-specific expression (see "Methods"), obtaining 308 expressed genes. Those genes included at least 38 transcription factors and genes with functions related to grain size and yield according to Gupta et al. (2020). The most promissing candidates taking into account the expression pattern and the predicted function are shown in Table 4.

## Discussion

The aim of the present study was to identify in a panel of wheat landraces, new genomic regions associated with key breeding traits, including grain traits, yield-related traits and phenological traits.

### Spanish bread wheat landraces present a wide range of phenotypic diversity

The phenotypic diversity of any collection of accessions is the limiting factor that will determine the chance to identify novel MTAs when conducting a GWAS. Thus, a successful study requires a collection as diverse as possible, but that at the same time is adapted to the target environment. For this analysis, a total of 189 Spanish bread wheat landraces, selected from 522 accessions to capture the available diversity regarding to collection site data (altitude, longitude, latitude) and morphological spike traits (Pascual et al. 2020a) have been characterized during five different seasons. Previous studies have pointed out the high degree of genetic diversity harboured by Spanish bread wheat landraces, highlighted, for example, by the high and novel allelic variability for prolamines (Giraldo et al. 2010; Ruiz et al. 2002). Moreover, Pascual et al. (2020b) have determined that the genetic diversity of this collection has not been included in the bread wheats currently cultivated in the country. When this collection was characterized at phenotypic level, this genetic diversity was translated into a wide range of phenotypic variation (i.e. grain traits variation shown on Table 1, Fig. 4d). It should be noted that variation is greater than that found in other landraces collections. For example, TKW presented a range of 25 gr in the season 2017–2018 (the wettest one) (Table 1), which is higher than that found in Asian landraces (according to Lopes et al. 2015). For yield related traits, such as PH, the range of variation was around 60 cm in all seasons (Table 1), similar to that found in a collection of Spanish durum wheat landraces (Giraldo et al. 2016). That is expected, as landraces precede the Green Revolution during which dwarfing genes were fixed, thus present higher variability than modern cultivars. Regarding phenological traits, differences in the latitude of landraces collection sites are typically related with diversity in vernalisation and photoperiod genes (Royo et al. 2020). The collection includes only Spanish accessions, however a range greater than a month was found for DH in all seasons (Table 1). This high phenotypic diversity has been also detected in Spanish durum wheat landraces (Giraldo et al. 2016), and it is probably due to the diverse environmental conditions found in Spain. Indeed, landraces were grown from cold sub-humid areas in the northern parts of Spain to warm semi-arid regimes in the southeast (Gadea 1954), in basic or neutral soils in the Centre and East, and acid soils in the western regions (Reuter et al. 2008).

### LD along the genome can be linked to the available genomic diversity

Linkage disequilibrium, the basis for association mapping, is mainly affected by historical recombination, allele frequency and selection in a natural population (Alqudah et al. 2020). In this work, LD and LD decay were evaluated. An average *r*2 = 0.06 for the whole genome was found, which is similar to the value obtained in other wheat landraces (Hanif et al. 2021). This low linkage disequilibrium is reflecting the lack of identity by descent, as the accessions predate the Green Revolution and thus do not share common parents in their pedigree and guarantees a high level of resolution when performing association analysis.

Comparing the different homoeologous genomes, it was found that, as previously described, the number of paired makers in LD was the lowest for the D genome and LD decay was also slower than for the A and B genomes (Pang et al. 2020; Jung et al. 2021) (Table S2). This might be due to the reduced genetic diversity of the D genome as a consequence of its relatively recent incorporation to bread wheat (IWGSC 2014). When focus was set at chromosome level, LD was the lowest at chromosome 7D and highest at chromosome 4B, however both chromosomes harbour a similar number of polymorphic markers (146 and 145 respectively) (Table S2). In this case, the difference does reflect the lower genetic diversity (Hs) at the centromeric region of chromosome 4B detected by Pascual et al. (2020b).

Finally, HAPLOVIEW software (Barrett et al. 2005) was employed to estimate the number of independent tests that could be performed with the selected molecular markers. The 4856 high quality SNP markers allowed to perform 4476 independent tests, a reduction of 7.8%, clearly lower than in other studies. For example, Rufo et al. (2021) genotyped their landraces with the Illumina Infinium 15K Wheat SNP Array, and from 10,090 high quality SNPs only considered 3696 to by independent. This fact indicates that the selected markers do not provide redundant information.

### GWAS in a collection of Spanish landraces uncover novel yield related MTA-QTLs

An association analysis combining the phenotypic data (11 traits in five different environments) from the highly diverse collection of landraces (189 accessions), and the set of high-quality SNP markers, considering the genetic structure (Pascual et al 2020b) identified a total of 881 Marker Trait Associations involving 434 markers across the genome (Fig. 2). Later, the genomic intervals (MTA-QTLs) that should contain the causal polymorphisms responsible of the phenotypic variance explained by the associated marker were defined according to LD. We identified 366 MTA-QTLs (Fig. 3 and Table S3), each of them associated with an average of 1.77 traits (ranging from 1 to 7 traits) and including an average of 1.35 markers, as expected considering the lack of redundancy found for the selected SNPs. MTA-QTLs were detected in all the wheat chromosomes; the A genome had the highest number of associations (152 MTA-QTLs) as previously described (Ain et al. 2015; Godoy et al. 2018; Khan et al. 2022), followed by the B (144) and D (62) genomes. Chromosome 5A, known for harbouring several genes affecting phenology and yield (Kato et al. 2000), included the highest number of MTA-QTLs (35) despite non-being the largest chromosome. In summary, our study revealed a large number of genomic regions implicated in key breeding traits, probably due to the wide agroclimatic diversity found in the Iberian Peninsula (Gadea 1954; Reuter et al. 2008). Moreover, several studies including landraces have previously shown the potential of those locally adapted accessions to reveal new associations, as example, Rahimi et al. (2019) and Rabieyan et al. (2022) analysed a collection including one hundred Iranian modern varieties two hundred Iranian landraces and detected 394 and 257 respectively.

To target which of the detected MTA-QTLs uncover novel associations with yield and yield related traits, the previous identified genes controlling the analysed traits and the most recent Meta-QTLs studies that best summarize the available information (Cao et al. 2020; Liu et al. 2020; Yang et al. 2021; Ma et al. 2022) were compared with the obtained results. Already known associations for grain and yield traits were validated in the present study, such as MTA-QTL 4A.182 (4A from 612.6 to 614 Mb) that includes the cell invertase *TaCWI* associated with kernel weight and grain number per spike (Jiang et al. 2015), MTA-QTL 5A.200 ( 5A from 49.59 to 136 Mb) that harbours *TaSnRK2* a protein kinase controlling yield related traits (Ur Rehman et al. 2019), MTA-QTL 6A.267 (6A from 230.15 to 285.56 Mb) that contains the widely studied *TaGW2* controlling grain size (Su et al. 2011) or MTA-QTL 7B.338 (7B from 68.15 to 71.35 Mb) inside which is located *TaSUS1* associated with TKW (Hou et al. 2014). Regarding phenological traits, four MTA-QTLs close by or including the well-known genes were detected; *PPD-D1* (Welsh 1973) (for MTA-QTL 2D.127 21–32Mb), *TaELF3-1DL* homolog to Early Flowering from Arabidopsis (Wang et al. 2016a) (for MTA 1D.63 483–486.34Mb), *VRN-B2* (Yan et al. 2004) (for MTA-QTL 4B.213, 655–670Mb), and *VRN-A1* (Yan et al. 2003) (for MTA-QTL 5A.215, 588–590Mb) for which it is already known the analysed set of landraces presents polymorphism (Pascual et al. 2020b). Then, the MTA-QTLs that to our knowledge are close by or include genes or QTLs previously identified were filtered out. The analysis revealed more than 150 considered novel associations, as were not included in the most recent Meta-QTLs analysis (Cao et al. 2020; Liu et al. 2020; Yang et al. 2021; Ma et al. 2022). New MTA-QTLs were identified for most of the characterized traits (except SpIN), moreover non-previously described associations could be found in all the chromosomes. Those results reflect the unexplored genetic diversity harboured by the bread wheat Spanish landraces (Pascual et al. 2020b), and are in accordance with those of Giraldo et al. (2016), where a GWAS in Spanish durum wheat landraces revealed mainly novel associations. Even though landraces present lower yields compared to modern cultivars under optimal conditions, they usually present more stable yields under harsh environments (Zeven 1998). Thus, those novel associations might include key genes that will enhance breeding programmes considering the actual climate change scenario. To look for putative candidate genes underlying the novel associations, we identified the annotation of gene located closest to the most significant marker inside each novel MTA-QTL. More than ten transcription factors and plant hormone related genes were identified.

### Dissection of high-density MTA-QTLs genomic regions identified new putative genes related with wheat yield

Genomic regions harbouring associations to several traits are especially useful for breeding, as they allow selecting for multiple traits. In this work, we identified 33 high density QTLs regions, associated with more than four traits and non-related with days to heading. One fifth of those regions were located on chromosome 5A, which again highlights the key role of this chromosome in adaptability and yield related traits control (Barabaschi et al. 2015). As expected, considering the high number of traits associated with them, most of those regions had been previously described (Cao et al. 2020; Liu et al. 2020; Yang et al. 2021; Ma et al. 2022). However, according to these studies some of the Meta-QTLs include hundreds of Mb. The present study helps to narrow the genomic interval that may include the causal genes, thus facilitates the search of putative candidates. For example, Liu et al. (2020) identified a Meta-QTL for TKW for chromosome 7B (size 65 Mb), which co-localized with R7B.2 whose size is just 2.27 Mb and includes the genes *TraesCS7B03G1112900* and *TraesCS7B03G1114600* two promising candidates. Moreover, one of those regions R5A.3 (described also by Cao et al. 2020; Yang et al. 2021) originally was linked to DH, DM (Table S4), and included the gene *VRN-A1* (589Mb according to *Triticum aestivum* genome REFseq v2.1). After taking into account the effect of DH, the region was reduced by 2 Mb, to the interval from 584.83 to 588.52 Mb at chromosome 5A, and remained associated to Ar, Perim, Majell and TKW (Table 3). This suggest that the already described link between DH and TKW (Giraldo et al. 2016) might be due to linkage disequilibrium between *VRN-A1* and another gene affecting grain weight. An ancestral recombination, that might have taken place during the selection of Spanish bread wheat landraces, may have helped to detect this link and suggests that exists an underexploited gene in this interval. Besides, to our knowledge four of the targeted regions, located on chromosomes 1A, 3A and 3B, have not been previously linked to the studied traits (Table 3). For one of them, R1A.1 the closest gene to the most significant MTA is *TraesCS1A03G0122300* a RING/U-box superfamily protein. It is well known that RING/U-box ubiquitin ligases play a role in plants growth and development, as well as in regulating the response to different stresses (Serrano et al. 2018). Actually in wheat and rice several studies have identified U-Box ubiquitin ligases as responsible for the regulation of grain related traits (Song et al. 2007; Wang et al. 2022; Brinton et al. 2018).

Finally, the focus was set on the six genomic regions that were linked to Ar, Perim, Majell and TKW in at least three seasons (stable QTLs) (Fig. 2). The total genes (413) included on these regions were carefully analysed to detect putative candidates, annotation as well as in silico expression analysis allowed the identification of the 15 most promising genes (Table 4).

Inside R1B.2 two putative genes coding for MYB transcription factors (*TraesCS1B03G0803000* and *TraesCS1B03G0817400*) were identified. This family of transcription factors is involved in different physiological and biochemical processes, including control of cell development and cell cycle, hormone synthesis, and signal transduction (Dubos et al. 2010; Feller et al. 2011). Moreover, according to KnetMiner database (Hassani-Pak et al. 2021) those genes regulate grain size related genes. Besides the gene *TraesCS1B03G0827400* that codes for a ubiquitin ligase whose link to yield has already been described was also selected inside this region based on its predicted expression (Wang et al. 2016b).

The second region R2B.6 (Fig. 4) located at the end of chromosome 2B (752.7–757.03) was considered the most promising one, as harboured the most significant MTA for TKW in three seasons, which produced also the greatest effects on grain size (Fig. 4d). It included a RNA binding protein (*TraesCS2B03G1383200*), as well as, the transcription factor *TraesCS2B03G1382600* with a high homology to rice *ILI1* gene. This rice gene, according to Zhang et al. (2009), acts as a positive regulator of cell elongation and plant development, having a positive role in leaf bending. Moreover, the rice gene *ILI6* from same family, plays a key role in determining rice grain length (Heang and Sassa 2012).

In chromosome 3B two regions were highlighted. R3B.1 (12.61–16.82 Mb), in which four candidates were selected, *TraesCS3B03G0058000*, a putative Cytochrome P450 highly expressed in spikelets, and three kinases *TraesCS3B03G0054900*, *TraesCS3B03G0055300* and *TraesCS3B03G0055900*. The kinases presented a high homology to Leucine-Rich-Repeat (LRR) receptor kinases SERK2, SERK4 and BAK1 from rice, involved in the regulation of plant growth through the brassinosteroid signalling pathway (Li et al. 2009; Park et al. 2011). The second region R3B.2 (249.38–258.96Mb), harboured an expressed NAC domain protein (*TraesCS3B03G0504300*) whose role in developmental process in widely known (Olsen et al. 2005), and a WD-repeat protein (*TraesCS3B03G0496600*) that codifies for a TOPLESS-related protein. The TOPLESS proteins play multiple roles throughout plant development (Causier et al. 2012; Oh et al. 2014).

On chromosome 5A (590.25–596.91Mb), two kinase proteins (*TraesCS5A03G0945200* and *TraesCS5A03G0956000*) were found within the R5A.4 region. The first one is highly similar to *BRI1* a Brassinosteroid LRR receptor kinase from rice, which increases the biomass and grain production in this species (Morinaka et al. 2006). The second one, codes for a Sucrose non-fermenting-1-related protein kinase 2.8 (SnRK2), an orthologue of *AT3G50500* Arabidopsis protein, involved in the abscisic acid signalling.

The last region R7B.1 (688.21–69.48) harboured *TraesCS7B03G1114600* and *TraesCS7B03G1112900*, an ubiquitin and F-box family protein, respectively, both highly expressed in spike and grains.

In summary, the present study of a collection of Spanish bread wheat landraces highlighted the high phenotypic diversity of this collection and identified more than 350 MTA-QTLs, including at least 150 novel ones. Those MTA-QTLs allowed the targeting of 33 high dense QTL regions in the genome, that remained associated to at least four traits after considering the effect of days to heading. Finally, taking into account the importance of detecting stable QTLs, six regions associated to several grain traits and TKW in at least three environments were selected as the most promising ones to harbour targets for breeding. Moreover, the preliminary screening for candidate genes reported in this study provide a starting point for future analysis aimed at the identification and validation of wheat yield related genes.

### Supplementary Information

Below is the link to the electronic supplementary material.

Supplementary file1 (XLSX 57 KB)

Supplementary file2 (DOCX 13 KB)

Supplementary file3 (XLSX 100 KB)

Supplementary file4 (XLSX 13 KB)

Supplementary file5 (PDF 256 KB)

Supplementary file6 (PDF 360 KB)

Supplementary file7 (PDF 241 KB)

Supplementary file8 (PDF 283 KB)

## Data Availability

The datasets generated during and/or analysed during the current study are available as supplementary material (average values), or from the corresponding author on reasonable request (raw values).
