# Supplementary material for: Genome wide association in Spanish bread wheat landraces identifies six key genomic regions that constitute potential targets for improving grain yield related traits
Source: Theor Appl Genet. 2023 Nov 13;136(12):244. doi: 10.1007/s00122-023-04492-x (PMC10643358; doi:10.1007/s00122-023-04492-x)
Supplement: Supplementary file 2 — Supplementary file2 (DOCX 13 KB) [file 122_2023_4492_MOESM2_ESM.docx]

**Table S2. Summary of observed LD.** Linkage Disequilibrium was estimated among marker pairs per chromosome, marker pairs were considered in significant LD when p-value was lower than 0.001.

|  | **Total Marker Pairs** | **% Marker Pairs** | **Average r2** | **Significant Marker Pairs** | **% Significant Marker Pairs** |
| --- | --- | --- | --- | --- | --- |
| **1A** | 13272 | 6,18 | 0,071 | 3791 | 28,56 |
| **1B** | 12568 | 5,86 | 0,073 | 3294 | 26,21 |
| **1D** | 3919 | 1,83 | 0,037 | 488 | 12,45 |
| **2A** | 13856 | 6,46 | 0,070 | 3518 | 25,39 |
| **2B** | 16015 | 7,46 | 0,067 | 3963 | 24,75 |
| **2D** | 6471 | 3,01 | 0,054 | 957 | 14,79 |
| **3A** | 14474 | 6,74 | 0,066 | 3795 | 26,22 |
| **3B** | 15616 | 7,28 | 0,052 | 3260 | 20,88 |
| **3D** | 4023 | 1,87 | 0,032 | 398 | 9,89 |
| **4A** | 7424 | 3,46 | 0,049 | 1425 | 19,19 |
| **4B** | 5973 | 2,78 | 0,088 | 1618 | 27,09 |
| **4D** | 2475 | 1,15 | 0,031 | 233 | 9,41 |
| **5A** | 15124 | 7,05 | 0,056 | 3570 | 23,60 |
| **5B** | 13472 | 6,28 | 0,060 | 3384 | 25,12 |
| **5D** | 5014 | 2,34 | 0,048 | 726 | 14,48 |
| **6A** | 9067 | 4,22 | 0,048 | 1527 | 16,84 |
| **6B** | 13017 | 6,06 | 0,057 | 2996 | 23,02 |
| **6D** | 4775 | 2,22 | 0,033 | 491 | 10,28 |
| **7A** | 17020 | 7,93 | 0,060 | 3903 | 22,93 |
| **7B** | 12721 | 5,93 | 0,073 | 3458 | 27,18 |
| **7D** | 5925 | 2,76 | 0,030 | 562 | 9,49 |
| **Unknown** | 2425 | 1,13 | 0,020 | 155 | 6,39 |
|  |  |  |  |  |  |
| **Genome A** | 90237 | 42,04 | 0,061 | 21529 | 23,86 |
| **Genome B** | 89382 | 41,64 | 0,065 | 21973 | 24,58 |
| **Genome D** | 32602 | 15,19 | 0,039 | 3855 | 11,82 |
| **Total** | 214646 | 100,00 | 0,059 | 47512 | 22,14 |
