# Supplementary material for: Genome wide association in Spanish bread wheat landraces identifies six key genomic regions that constitute potential targets for improving grain yield related traits
Source: Theor Appl Genet. 2023 Nov 13;136(12):244. doi: 10.1007/s00122-023-04492-x (PMC10643358; doi:10.1007/s00122-023-04492-x)
Supplement: Supplementary file 5 — Supplementary file5 (PDF 256 KB) [file 122_2023_4492_MOESM5_ESM.pdf]

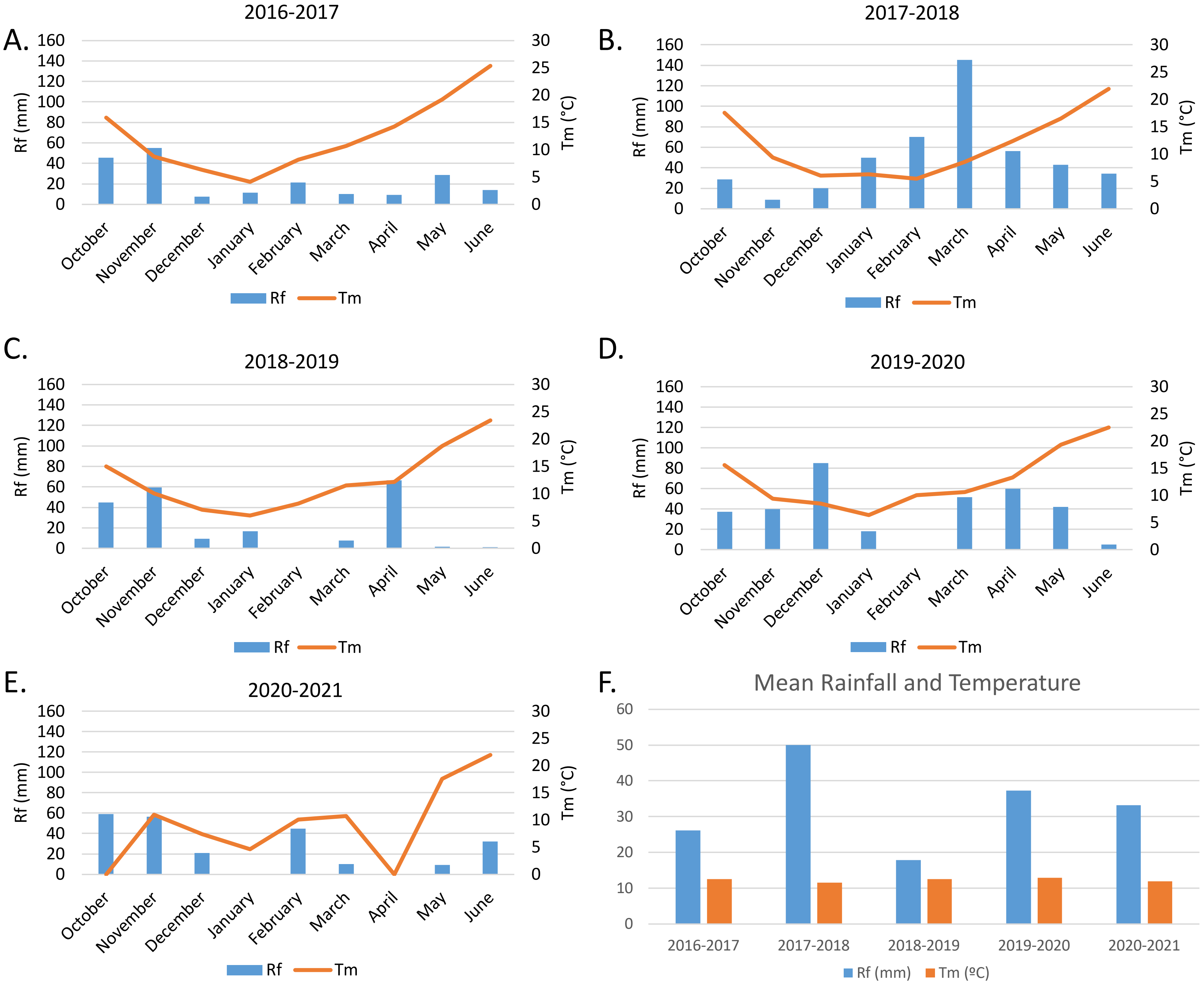

Fig. S1. Summary of climatic data during the phenotypic trials. Monthly rainfall (Rf, mm) in orange, and mean temperature (Tm, ºC) blue, recorded from October to July at the field trial sites during (A) 2016–2017, (B) 2017–2018, (C) 2018–2019, (D) 2019–2020 and (E) 2020-2021, and (F) mean temperature (Tm, ºC) and rainfall (Rf, mm) per season, both values indicated in the same axis.
