# Supplementary material for: Genome wide association in Spanish bread wheat landraces identifies six key genomic regions that constitute potential targets for improving grain yield related traits
Source: Theor Appl Genet. 2023 Nov 13;136(12):244. doi: 10.1007/s00122-023-04492-x (PMC10643358; doi:10.1007/s00122-023-04492-x)
Supplement: Supplementary file 6 — Supplementary file6 (PDF 360 KB) [file 122_2023_4492_MOESM6_ESM.pdf]

A

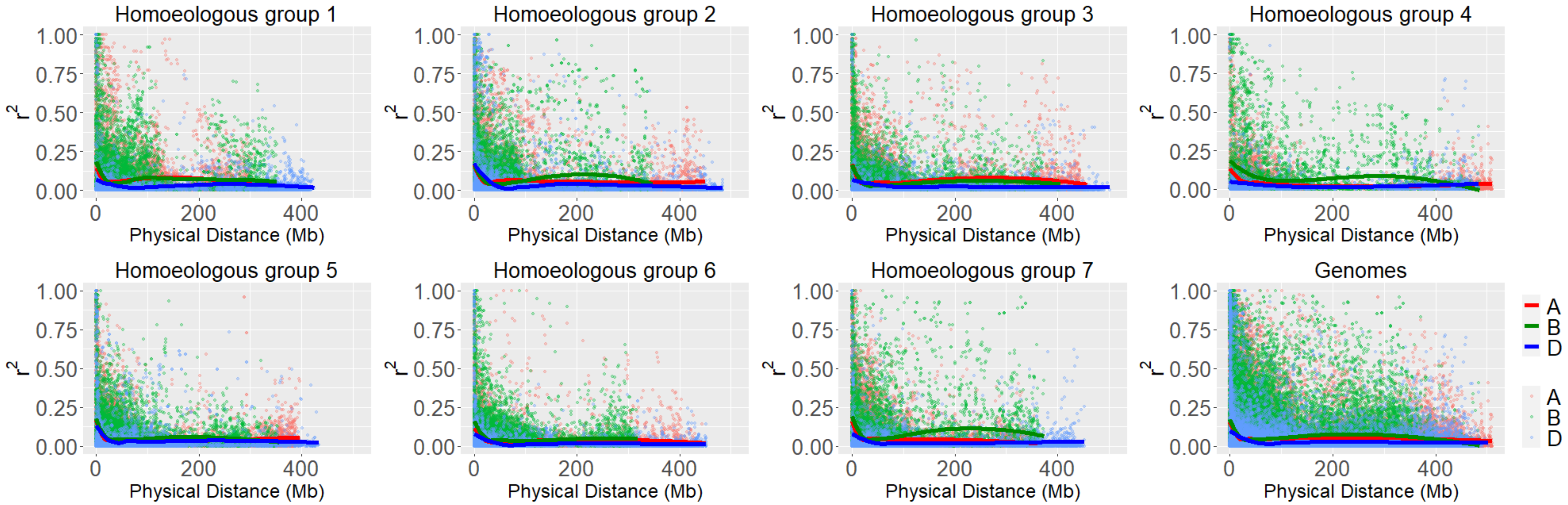

Fig. S2. LD (r2) decay. (A) along the chromosomes and full genome. Genome A in red, B in green and D in blue. (B) LD (r2) decay indicating half LD decay and half LD decay distance in the full genome.

B

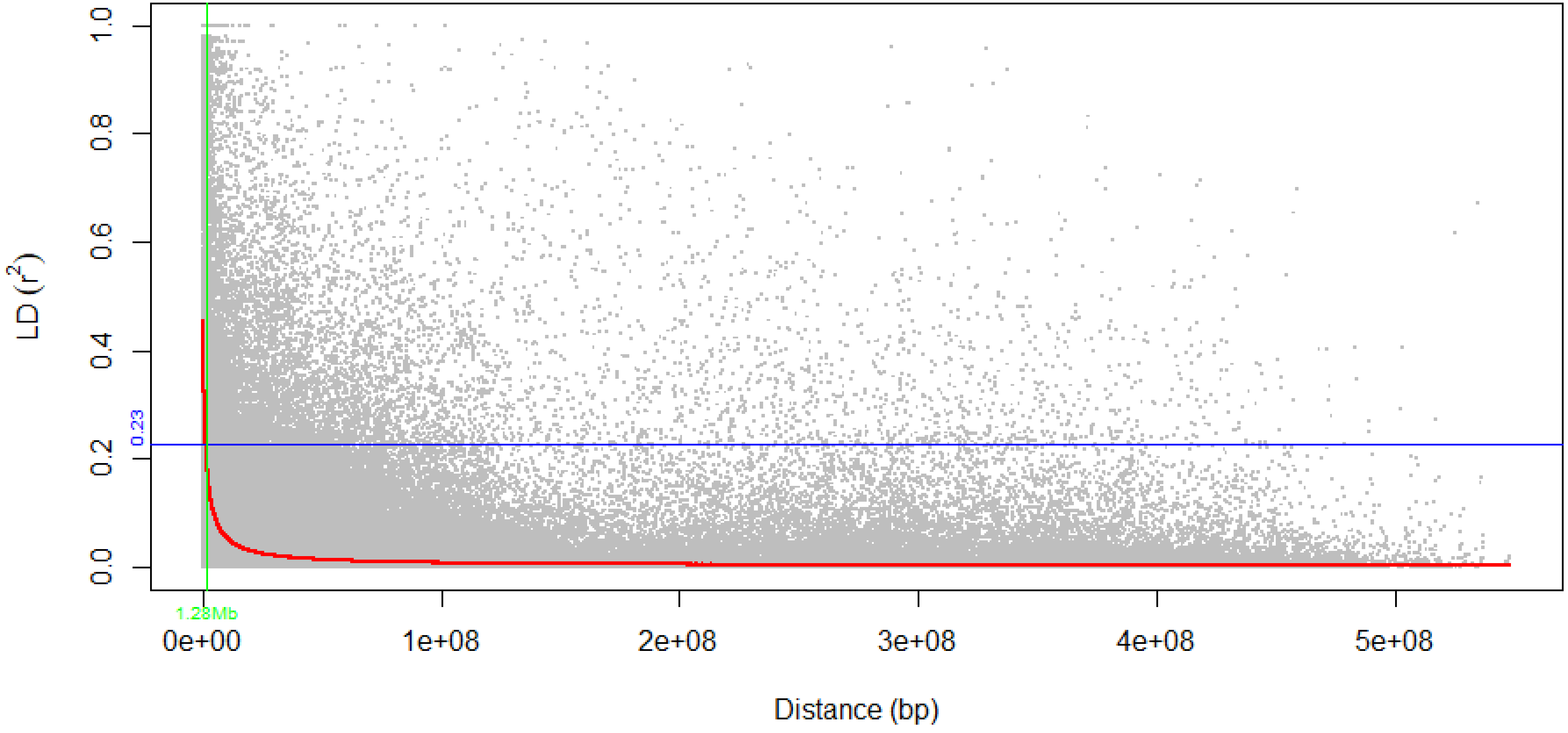

Fig. S2. LD (r2) decay. (A) along the chromosomes and full genome. Genome A in red, B in green and D in blue. (B) LD (r2) decay indicating half LD decay (blue) and half LD decay distance (green) in the full genome.
