# Supplementary material for: Genome wide association in Spanish bread wheat landraces identifies six key genomic regions that constitute potential targets for improving grain yield related traits
Source: Theor Appl Genet. 2023 Nov 13;136(12):244. doi: 10.1007/s00122-023-04492-x (PMC10643358; doi:10.1007/s00122-023-04492-x)
Supplement: Supplementary file 7 — Supplementary file7 (PDF 241 KB) [file 122_2023_4492_MOESM7_ESM.pdf]

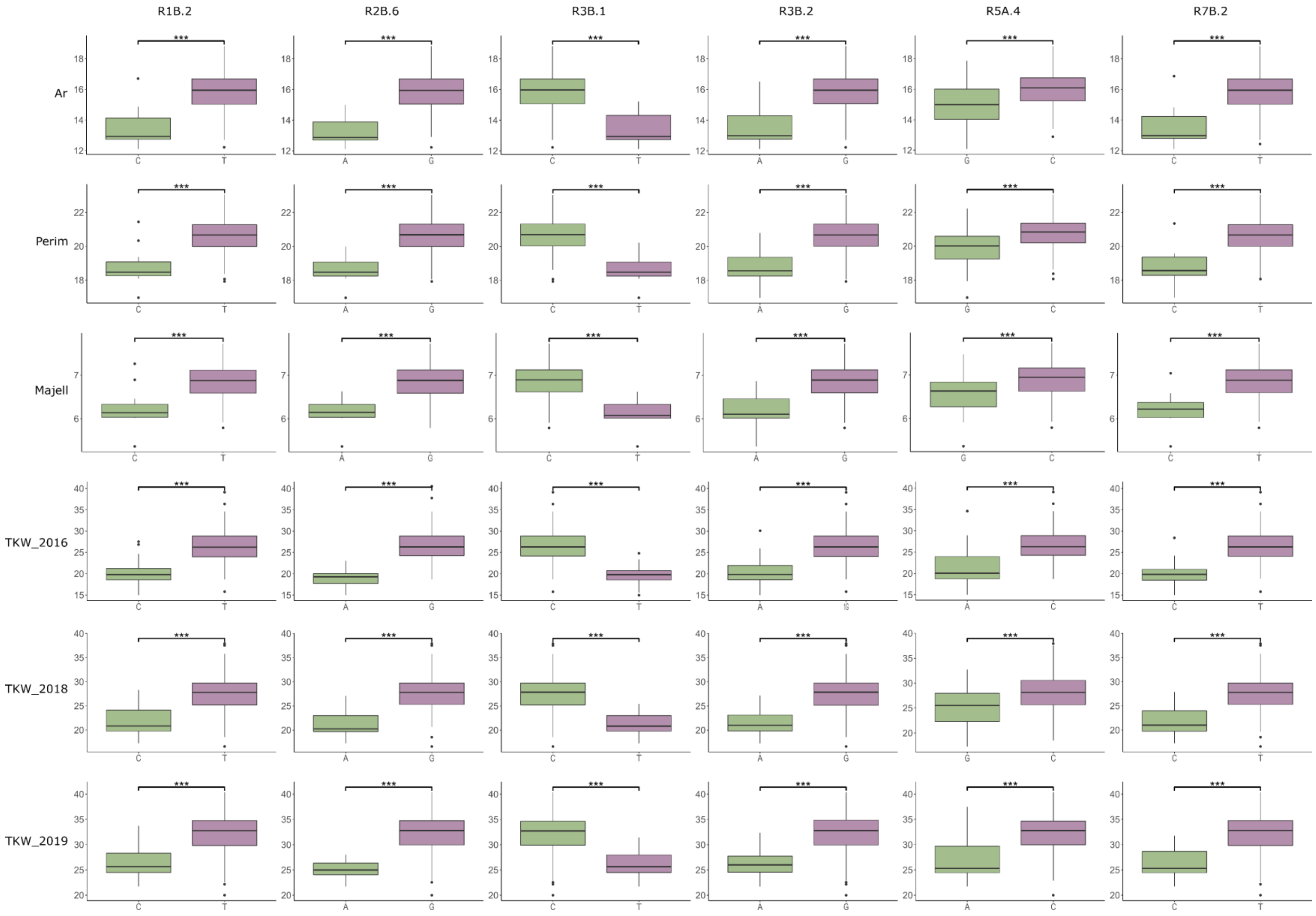

Fig. S3. Average trait value according to the allele carried by the accessions in the most significant MTA inside the six selected genomic regions. Area: Ar; Perimeter: Perim; Major Ellipse: Majell; Thousand Kernel Weight: TKW. *** represented a *p-value*<0,001.
