# Supplementary material for: Genome wide association in Spanish bread wheat landraces identifies six key genomic regions that constitute potential targets for improving grain yield related traits
Source: Theor Appl Genet. 2023 Nov 13;136(12):244. doi: 10.1007/s00122-023-04492-x (PMC10643358; doi:10.1007/s00122-023-04492-x)
Supplement: Supplementary file 8 — Supplementary file8 (PDF 283 KB) [file 122_2023_4492_MOESM8_ESM.pdf]

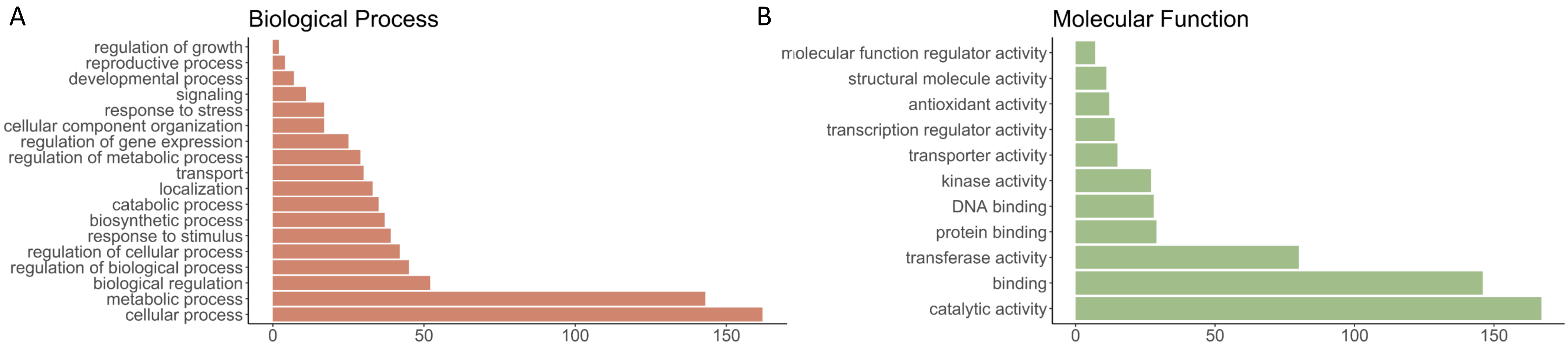

Fig. S4. Gene Ontology classification of genes within the selected regions. (A) Biological Process. (B) Molecular Function.
